# Cesarean Section in a Group 1 Pulmonary Hypertension Parturient Patient: A Case Report

**DOI:** 10.7759/cureus.63390

**Published:** 2024-06-28

**Authors:** Alexander Sumarli, Jeannie Choi, Vincent Wong, Nadia Aluzri, Lauren Pineda, Edgardo Reynoso, Kara Lodenkamp, Uoo Kim

**Affiliations:** 1 Anesthesiology, Loma Linda University School of Medicine, Loma Linda, USA; 2 Anesthesiology and Perioperative Medicine, Loma Linda University Medical Center, Loma Linda, USA

**Keywords:** hemodynamic management, extracorporeal membrane oxygenation, systemic lupus erythematosus, parturient, pulmonary hypertension

## Abstract

Severe pulmonary hypertension (PH) during pregnancy poses considerable challenges due to the physiological changes and increased cardiovascular demands. Close multidisciplinary management is essential throughout the peripartum period. The critical steps taken to provide anesthesia safely and successfully for a planned cesarian section are outlined, with special care for communication between the cardiothoracic surgery and obstetric team. A 31-year-old G3P1112 (three pregnancies, one term delivery, one pre-term delivery, one abortion, with two living children) patient with a history of systemic lupus erythematosus complicated by Group 1 PH presented to the operating room for a planned 34-week cesarean section. Pulmonary artery systolic pressure (PASP) was noted to be 68 mmHg at this time. Intravenous (IV) treprostinil at 8 ng/kg/min through a tunneled right subclavian line was initiated in her third trimester, and a day before her cesarean section, she was admitted for a lumbar epidural catheter placement. In the operating room, IV treprostinil was continued and a high-flow nasal cannula with inhaled nitric oxide at 20 ppm was initiated. A right internal jugular vein pulmonary artery catheter was placed for close monitoring of her pulmonary artery pressures, with a PASP reading of 64 mmHg at the start of the case. Femoral arterial and venous access was placed by the cardiothoracic surgery team for cardiopulmonary bypass standby. Intra-operative surgical analgesia was achieved by epidural lidocaine. A cesarean section was performed and was uncomplicated despite her post-delivery autotransfusion, where her PASP went as high as 89 mmHg. Uterine atony was managed with an oxytocin infusion. Epidural morphine was administered through the epidural catheter for post-operative analgesia. In the post-operative recovery room, her PASP was back down to baseline at 62 mmHg. The patient proceeded to have an uneventful postpartum hospital stay and was discharged home without any complications. While severe PH poses a challenge in the care of a parturient patient, safe and successful management may be achieved as outlined in this case report.

## Introduction

Pulmonary hypertension (PH) is a progressive systemic disease that is characterized by abnormally increased pressures within the pulmonary vasculature [1]. While the gold standard for diagnosis of PH is a mean pulmonary artery pressure of 25 mmHg or greater on right heart catheterization, a measurement of estimated pulmonary artery systolic pressure (PASP) of 35-40 mmHg or greater on echocardiography is also highly suggestive of the disease [1,2]. Although often asymptomatic at first, this initially innocuous abnormality quickly creates devastating downstream effects. If left unchecked, these consequences result in both high morbidity and mortality.

PH is divided into five groups, with Group 1 being pulmonary artery hypertension where the pulmonary arteries become narrowed and thickened from medical conditions such as systemic lupus erythematosus (SLE), idiopathic, or hereditary causes. Group 2 is PH due to left heart disease, Group 3 due to lung disease, Group 4 due to chronic thromboembolic PH, and Group 5 due to unknown causes. Our patient belongs in the Group 1 category with her PH caused by SLE. 

The effects of PH are mainly created by the proliferation of vascular endothelial cells and fibroblasts, which remodel the pulmonary vasculature over time [3]. This increased resistance over time creates chronic right heart strain [1,3]. As the right ventricle (RV) hypertrophies in order to compensate for the increased afterload of the pulmonary circulation, the structural changes of the RV bring on diastolic dysfunction [4]. The non-distensible myocardium constricts the inner chamber, preventing adequate blood filling and decreasing RV stroke volume [4,5]. Furthermore, if left untreated, the septal hypertrophy of the RV may bow into the left ventricle (LV), which will limit its preload by 10-20% [4,5]. It is clear that the consequences of PH have drastic structural effects on both sides of the heart, which over time will precipitate a right ventricular heart failure.

Unfortunately, in parturient patients, the physiologic changes compound the amount of stress on the heart because of the increased physiologic demand to raise cardiac output and deliver blood to both the mother and fetus [2]. During normal pregnancy, the plasma volume can increase by 50% and cardiac output can increase by up to 40% by 37 weeks of gestation [6]. However, in PH, the remodeling of both the pulmonary vasculature and RV myocardium impairs the body’s ability to accommodate these physiologic changes [2,7]. This can result in right-sided heart failure and limited preload delivery to the left side of the heart, which precipitates hypotension and even death. Recent literature shows that although PH in pregnancy is uncommon (1/100,000), maternal and fetal mortality rates can range from 11-50% because of this sudden hemodynamic compromise [2,7,8]. Coupled with the cardiovascular effects of anesthesia, these patients can be difficult to manage. This clinical case presents a multidisciplinary approach in anesthetic and hemodynamic management for a parturient patient with PH.

## Case presentation

A 31-year-old G3P1112 (three pregnancies, one term delivery, one pre-term delivery, one abortion, with two living children) patient with a history of SLE complicated by Group 1 PH presented for a planned 34-week (by the last menstrual period) cesarean section in the operating room.

**Figure 1:**
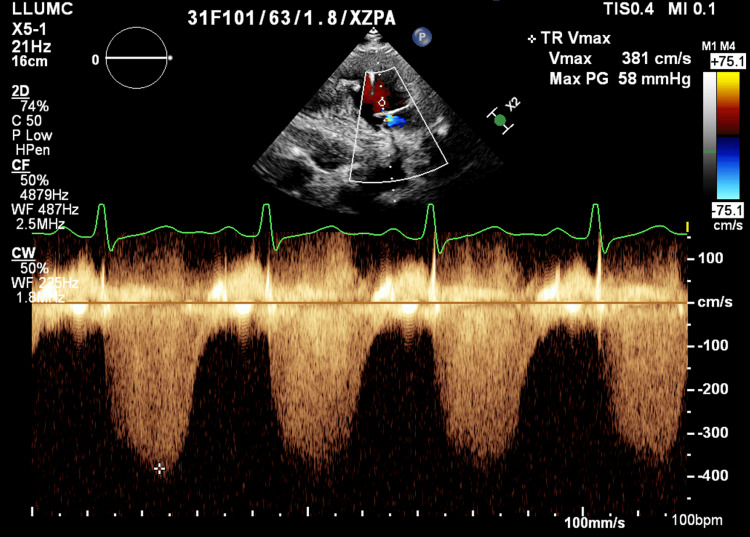
Transthoracic Echocardiography Estimating Pulmonary Artery Systolic Pressure TR Vmax: Tricuspid regurgitation max velocity; Max PG: pressure calculated by max velocity

The patient’s diagnosis of SLE was made at the age of 18, with subsequent diagnosis of pulmonary hypertension at age 21. She was previously on multiple vasodilators including riociguat, ambrisentan, selexipag, and treprostinil. Despite medication compliance, the patient continued to have worsening PH, with symptoms including dyspnea with walking up stairs and lower extremity swelling. During her prepartum period, the patient was followed by her pulmonologist and rheumatologist. Within the first trimester, the patient stopped taking her medications given significant nausea and vomiting as a side effect of pregnancy, and her PASP was estimated to be above 58 mmHg by transthoracic echocardiography, with a moderate decrease in right ventricular (RV) systolic function at that time (Figure 1). She established care with her high-risk obstetrics physician by the late first trimester, and the patient was admitted to initiate intravenous (IV) prostacyclin for control of her PH. Oral tadalafil at 40mg was started as her nausea and vomiting improved in her second trimester. She continued to be on rituximab for her SLE throughout pregnancy. Towards her third trimester, IV treprostinil at 34ng/kg/min was started through a right tunneled subclavian vein placed by interventional radiology. 

**Figure 2:**
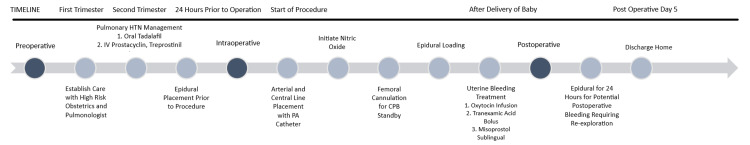
Timeline of Events PA: Pulmonary artery; CPB: cardiopulmonary bypass

One day prior to the cesarean section, the patient was admitted to the obstetrics service for placement of a lumbar epidural catheter by the obstetric anesthesiologist on call. The patient arrived at the operating room the next day on continuous IV treprostinil and epidural catheter in place. A high-flow nasal cannula with 20 ppm inhaled nitric oxide was initiated. The left radial arterial line was placed followed by the placement of a left internal jugular vein multi-lumen access catheter (MAC) central line. Pulmonary artery catheter (PAC) placement through the MAC port was unsuccessful due to resistance from the tunneled right subclavian line. A second right internal jugular MAC was placed with successful PAC placement, verified by transthoracic echocardiography. Initial PA pressure upon PAC placement was 78/36 mmHg. Once sterile drapes were up, the left femoral vein and right femoral artery were cannulated by cardiothoracic surgery in case of hemodynamic collapse during delivery necessitating cardiopulmonary bypass. Intra-operative surgical analgesia was achieved by 5mL increments of 2% lidocaine administered through her epidural over 15 minutes for a total dose of 400mg. Surgical analgesia was confirmed with loss of sensation and pain as tested by instrument skin pinch up to thoracic level T6 dermatome. A cesarean section was performed and the baby was delivered shortly thereafter, with an Apgar score of 9. Tranexamic acid bolus of 1 gram and oxytocin (40 units) was infused at a starting constant rate of 100 mL hourly, with subsequent increases in the infusion rate up to 250 mL/hr titrated by the patient's PA pressure and blood pressure. Methylergonovine and carboprost were contraindicated due to pulmonary hypertension and were not given. Sublingual misoprostol (800 mcg) was also administered and uterine tone was adequately achieved (Figure 2).

Estimated blood loss throughout the case was 500 mL. Uterine and skin closure was achieved using an additional 5 mL of 3% chloroprocaine. Autotransfusion post-delivery did show an increase in systolic PA pressures up to 89 mmHg but did not necessitate inotropic or vasodilator support as the patient remained asymptomatic and hemodynamically stable. Patient’s blood pressures and PA pressures were maintained within 10-20% post-operatively and post-operative analgesia was obtained with 3 mg of preservative free morphine through her epidural catheter. The rest of her post-operative hospital stay was uneventful and the patient was discharged home without further complications.

## Discussion

PH in pregnancy presents significant challenges due to the physiological changes and increased cardiovascular demands associated with pregnancy, which can lead to serious complications such as right ventricular failure. This is especially critical during cesarean sections, where anesthetic and hemodynamic management requires careful consideration to optimize both maternal and fetal conditions. This case report illustrates several key points regarding the perioperative and intraoperative management of such patients.

The patient presented in this case report had a complex medical history, as she required concurrent management of SLE and severe PH, which complicated her prenatal course. Her previous experience with various vasodilator medications, which didn’t effectively control her pulmonary arterial pressure, along with her limited compliance due to nausea and vomiting, made managing PH during pregnancy particularly challenging. The multidisciplinary approach is essential for optimizing the condition of such patients throughout their pregnancy [2,7,9]. This was evident in this case, where close monitoring by various specialists and collaboration between pulmonologists, rheumatologists, high-risk obstetricians, cardiothoracic surgeons, and anesthesiologists allowed for timely adjustments to the patient’s medical regimen. This also allowed for a jointly made decision to proceed with a cesarean section with epidural anesthesia, with cardiopulmonary bypass on standby. As a result, there was adequate control of her symptoms and PA pressures throughout her pregnancy and during her intraoperative care.

The mode of delivery significantly influences both maternal and fetal outcomes. Cesarean section is often the preferred mode of delivery for patients with PH because it can mitigate several hemodynamic risks associated with vaginal delivery [10,11]. First of all, the pain experienced during labor can activate the sympathetic nervous system, leading to tachycardia and an increase in both systemic and pulmonary vascular tone [10]. Valsalva maneuver during labor can increase intrathoracic pressure, reducing venous return and preload, and labor may also trigger vasovagal responses, further decreasing venous return [10]. In addition, labor can induce hypoxia, hypercarbia, and acidosis which can increase PA pressure leading to right ventricular failure exacerbation [10,12]. Moreover, vaginal delivery involves autotransfusion of blood from the contracting uterus and the movement of peripheral edema from the extravascular space into the systemic vasculature, which can lead to sudden shifts in maternal blood volume after delivering the fetus [10]. A planned elective cesarean section, on the other hand, can help prevent the above complications associated with labor and provide a more controlled and predictable environment for delivery. When planned and performed before the onset of labor, preferably around weeks 34-36, cesarean section enables multidisciplinary planning and allows anesthesiologists to prepare anesthetic strategies to minimize the risk of hemodynamic instability [10].

The choice of anesthesia is another important consideration for cesarean sections in parturient patients with PH. Although the anesthesia plan should be individualized, epidural anesthesia is generally preferred over spinal and general anesthesia. The risk of sympathectomy, also commonly termed “high-spinal”, with spinal anesthesia may carry a higher risk in this population. Neuraxial anesthesia can cause an abrupt drop in systemic vascular resistance, venous return, and cardiac output [9]. Therefore, epidural anesthesia with gradual incremental doses or low-dose combined spinal-epidural anesthesia, with careful monitoring, are considered the most effective approaches [9,10]. A systematic review covering publications from January 1997 to September 2007 indicated that the mortality rate was higher in parturient patients who received general anesthesia compared to those who received neuraxial anesthesia [12]. This finding may be attributed to various factors (in addition to the risk of a difficult airway), some of which include the physiological responses to general anesthesia. Hemodynamic changes in the pulmonary vasculature can occur particularly during laryngoscopy and endotracheal intubation, which can severely increase PA pressure [12-14]. Additionally, positive pressure ventilation can raise pulmonary vascular resistance (PVR) or RV afterload [12-14]. Furthermore, it is well known that anesthetics decrease cardiac contractility. In the context of PH, this can exacerbate an increase in end-diastolic volume and a reduction in LV preload, leading to a decrease in stroke volume and blood pressure [4]. Conversely, epidural anesthesia can help avoid the aforementioned complications. Numerous studies have demonstrated that neuraxial anesthesia techniques used during C-sections lead to successful outcomes, primarily due to their reduced impact on hemodynamics [8,9,15,16,17]. With the successful and timely placement of the epidural catheter and gradual loading of local anesthetics and opioids throughout the intraoperative and postpartum periods, our patient could achieve adequate analgesia and hemodynamic stability. Timeliness in the placement of an epidural catheter in accordance with the American Society of Regional Anesthesia neuraxial guideline, in anticipation of a possible bolus dosing of heparin in the setting of ECMO cannulation, is critical.

In managing parturient patients with PH undergoing cesarean delivery, the use of uterotonic agents warrants careful consideration due to its cardiovascular effects. Uterotonics, commonly used to promote uterine contraction after delivery to prevent postpartum hemorrhage, can decrease systemic vascular resistance and increase PVR, which can exacerbate cardiovascular collapse [18,19]. Among these agents, methylergonovine and carboprost are contraindicated in patients with PH because of their potential vasoconstrictive effects on pulmonary vasculatures [20,21]. Oxytocin and misoprostol are more acceptable choices for such patients, as their impact on pulmonary artery pressure is relatively low [21]. When oxytocin is administered in large concentrations or as a bolus dose, it can cause significant hypotension and tachycardia secondary to a decrease in systemic vascular resistance and venous return. Therefore, administering oxytocin via a slow infusion with continuous monitoring is recommended [10,20,22]. In this case report, the patient was given oxytocin in a low-dose infusion, which was gradually titrated under strict monitoring, including central venous pressures and PA pressures.

## Conclusions

Through careful planning and perioperative monitoring, it is possible to successfully manage a complex parturient patient with PH, achieving a favorable outcome despite the high risks involved. This case highlights key insights essential for effectively navigating the complex interplay of PH and pregnancy. It emphasizes the benefit of a multidisciplinary approach in both preoperative and intraoperative management. It also stresses the careful selection of delivery mode and timing, particularly the advantages of cesarean section in providing a controlled environment to mitigate hemodynamic risks. Furthermore, tailored anesthesia strategies, particularly the use of epidural anesthesia, are crucial for maintaining hemodynamic stability during delivery. Additionally, the judicious use of uterotonic agents is essential, favoring options such as oxytocin that have milder effects on systemic as well as pulmonary hemodynamics. This case contributes to the expanding literature supporting a structured, team-based, and patient-centered approach to managing such high-risk pregnancies involving C-sections. It underscores the need for ongoing research and discussion to refine and improve management strategies for such high-risk patient populations, thereby optimizing outcomes in similar future cases.
